# Using a Novel Connected Device for the Collection of Puffing Topography Data for the Vuse Solo Electronic Nicotine Delivery System in a Real-World Setting: Prospective Ambulatory Clinical Study

**DOI:** 10.2196/49876

**Published:** 2023-10-30

**Authors:** Robert Underly, Gary M Dull, Evan Nudi, Timothy Pionk, Kristen Prevette, Jeffrey Smith

**Affiliations:** 1 Reynolds American Incorporated Services Company Winston-Salem, NC United States; 2 R Street Institute Washington DC, DC United States

**Keywords:** topography, electronic cigarette, e-cigarette, electronic nicotine delivery system, ENDS, ambulatory puffing, use behavior, sessions, mobile phone

## Abstract

**Background:**

Over the last decade, the use of electronic nicotine delivery systems (ENDSs) has risen, whereas studies that describe how consumers use these products have been limited. Most studies related to ENDS use have involved study designs focused on use in a central location environment or attempted to measure use outcomes through subjective self-reported end points. The development of accurate and reliable tools to collect data in a naturalistic real-world environment is necessary to capture the complexities of ENDS use. Using connected devices in a real-world setting provides a convenient and objective approach to collecting behavioral outcomes with ENDS.

**Objective:**

The Product Use and Behavior instrument was developed and used to capture the use of the Vuse Solo ENDS in an ambulatory setting to best replicate real-world use behavior. This study aims to determine overall mean values for topography outcomes while also providing a definition for an ENDS use session.

**Methods:**

A prospective ambulatory clinical study was performed with the Product Use and Behavior instrument. Participants (n=75) were aged between 21 and 60 years, considered in good health, and were required to be established regular users of ENDSs. To better understand use behavior within the population, the sample was sorted into percentiles with bins based on daily puff counts. To frame these data in the relevant context, they were binned into low-, moderate-, and high-use categories (10th to 40th, 40th to 70th, and 70th to 100th percentiles, respectively), with the low-use group representing the nonintense category, the high-use group representing the intense category, and the moderate-use group being reflective of the average consumer.

**Results:**

Participants with higher daily use took substantially more puffs per use session (6.71 vs 4.40) and puffed more frequently (interpuff interval: 32.78 s vs 61.66 s) than participants in the low-use group. Puff duration remained consistent across the low-, moderate‑, and high-use groups (2.10 s, 2.18 s, and 2.19 s, respectively). The moderate-use group had significantly shorter session lengths (*P*<.001) than the high- and low-use groups, which did not differ significantly from each other (*P*=.16).

**Conclusions:**

Using connected devices allows for a convenient and robust approach to the collection of behavioral outcomes related to product use in an ambulatory setting. By using the variables captured with these tools, it becomes possible to move away from predefined periods of use to better understand topography outcomes and define use sessions. The data presented here offer a possible method to define these sessions. These data also begin to frame international standards used for the analytical assessments of ENDSs in the correct context and begin to shed light on the differences between standardized testing regimens and actual use behavior.

**Trial Registration:**

Clinicaltrials.gov NCT04226404; https://clinicaltrials.gov/study/NCT04226404

## Introduction

### Background

The use of electronic nicotine delivery systems (ENDSs) has increased greatly over the last decade; however, very little is known about how consumers are using these products [1]. This limitation is mainly driven by ineffective or antiquated methodologies for capturing consumer ENDS behavior. Most studies related to the use of ENDS products have focused on use in a confined location in which participants are given a set period of time to use the product or are allowed ad libitum use, but the duration of the time the product can be used is controlled [2-6]. These types of methodologies provide only a snapshot of use behavior at a specific moment in time. Some studies have attempted to capture use over time [7-9], but only a few have been able to effectively generate cumulative time-series data sets. The importance of understanding use in an ambulatory environment (ie, a setting outside of a clinical laboratory that allows real-world naturalistic ENDS use) has broad implications because it relates to accurately understanding the testing paradigms used to assess ENDSs [7-9] and forming an understanding of how consumers can be effectively transitioned to less harmful products. The current methods to measure ENDS puffing topography can be lacking in certain aspects, such as being focused on the use of ENDSs in clinical laboratories and for a set period of time, which may not reflect natural use [6,10,11]. Some confined-use studies have methods similar to those of previous cigarette topography research studies [12,13], and 1 advantage of these methods is that some of them (eg, the use of video recording) can be performed without any additional apparatus attached to the ENDS products [14], or they can be conducted with the use of inexpensive commercially available equipment [15]. Although these confined methods may work for cigarettes that have a definite start point and a finite end point, ENDS products are typically used more spontaneously throughout the lifetime of a cartridge or battery. Restraining ENDS use to a specific period of time may not represent what a typical use session would look like for a specific user in terms of puffing characteristics, such as puff duration, the length of and time between discrete sessions of ENDS use, or the number of these discrete sessions during a prolonged period of use.

To improve on these previous studies, other studies have focused on methods that allow for more spontaneous ENDS use in a natural environment. However, these studies can also have their own limitations; for example, many of these topography studies used puffing measurement devices that fit between the mouthpiece of the ENDS product and the user, making them cumbersome to use compared with using the ENDS device alone. These measurement devices also require user or technician input (eg, a calibration step has to be undertaken before each use session) [9,16-20]. This has the potential to affect spontaneous user experience by affecting vapor output to the user. This could lead to less than natural use patterns because these factors may change how the ENDS device is used. Furthermore, many of these natural use studies have found large degrees of inter- and intraparticipant variability [20,21], with, for example, participant-specific SDs for puff duration ranging from 0.8 to 4.1 seconds and with similarly large variations for flow rate and puff volume [20]. In addition, mean puff durations ranged from approximately 1 second to 3.5 seconds among participants [20]. Although this variability could be natural among users, there is potential for it to be caused by the cumbersome nature of the topography measurement devices or other factors.

### A Novel Tool to Capture Puffing Behavior Data

**Figure 1:**
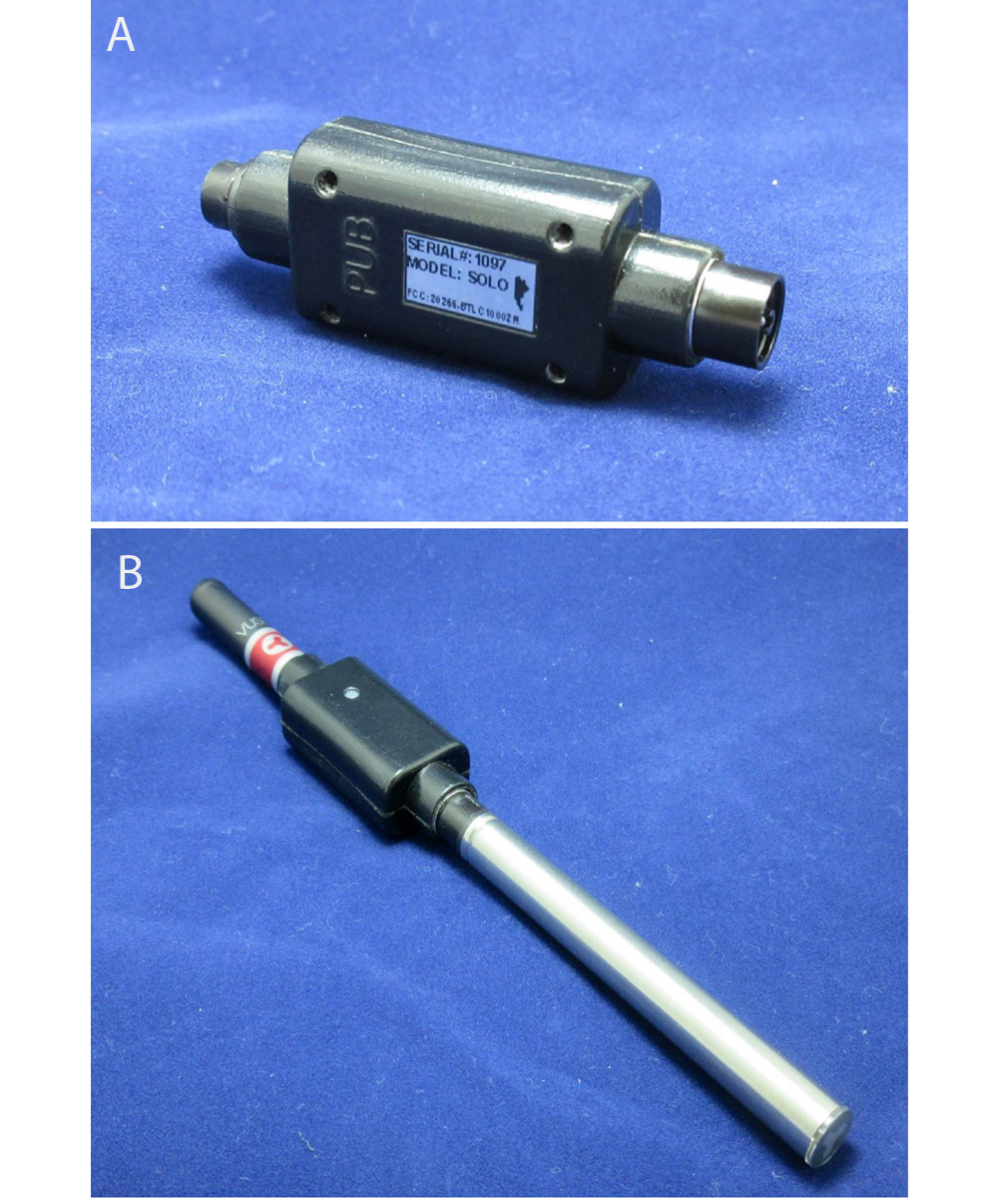
Photographic images showing the Product Use and Behavior (PUB) instrument. Images show (A) the PUB device alone and (B) the PUB device attached to the Vuse Solo electronic nicotine delivery system.

The Product Use and Behavior (PUB) instrument is a novel tool developed by RJ Reynolds Tobacco Company in partnership with Carolina Medical Electronics (CME). This instrument allows for the capture of puffing behavior data in both clinical and ambulatory settings with only minor modifications to user-product interactions and allows for cumulative time-series data to be captured [22]. The PUB instrument is a small, product-specific, and battery-powered attachment that fits between the ENDS battery and electronic liquid (e-liquid) cartridge that captures puff count, puff duration, and interpuff interval (IPI), as well as voltage and current data (Figure 1). Accompanying the device is a data capture and integration system that includes a mobile app for data collection, an integrated data transfer system, and a software package allowing for individual study setup and participant assignment. This study used this novel technology to capture cumulative time-series data during normal daily use in an ambulatory setting and sought to address the challenge of accurately capturing and understanding daily ENDS puffing topography and use patterns. This approach allowed us to frame traditional puffing topography end points such as the total number of puffs; individual puff durations; and the interval between puffs within the context of novel session-related end points, including puffs per session, time between sessions, sessions per day, and session length. Collecting puffing topography data in an ambulatory setting and framing the data in the context of sessions allowed for a more complete understanding of ENDS use patterns. By capturing and analyzing ENDS use patterns through *real-world* data collection methods, we can begin to better differentiate the potential public health impacts of ENDS use from those of other tobacco products.

## Methods

### Study Design and Participants

This was a prospective ambulatory clinical study conducted at a single site (Alliance for Multispecialty Research) in Knoxville, Tennessee, United States. The study was registered with ClinicalTrials.gov (NCT04226404).

Puffing topography and ENDS use data were collected in recruited users of closed-system ENDSs. Eligible participants were men and women (aged 21-60 y) in good health who were current regular users of closed-system ENDSs (ie, they self-reported the use of ≥2 ENDS cartridges per week in the past 30 d) as their primary form of tobacco- or nicotine-containing product use. Participants attempting to quit and those with exclusionary preexisting medical conditions that would preclude them from participation per the study protocol were excluded from the study. Individuals could also be excluded if it was deemed unsafe for them to participate by the principal investigator. Participants were enrolled directly after screening.

### Ethical Considerations

The study protocol, which included information concerning the Vuse Solo ENDS product, was approved by Advarra Inc (reference number: Pro00040690). The study was conducted in accordance with the ethical standards in the 1964 Declaration of Helsinki, relevant sections of the US Code of Federal Regulations (21 CFR part 50, part 54, part 56, and part 312), and the ethical principles embodied in the International Council for Harmonisation of Technical Requirements for Pharmaceuticals for Human Use Guideline for Good Clinical Practice (E6[R2]).

For this study participantss were compensated $200 USD for completing the screening visit, $70 for the completion of days 1-7 of the study, $285 for the completion of days 8-21 and $100 for the end of study visit. Participants were informed that their health data would be shared with the study doctor and may appear in study information provided to contract research organization (ICON). Data were anonymized and participants were informed that no identifying information would be collected beyond their written consent. Participants were informed that their study data could be used for additional secondary analysis, but that they could revoke permission to share their data at any point.

### Study Products

The study used the Vuse Solo closed-system e-cigarettes (RJ Reynolds Vapor Company). Seven flavors of e-liquids were available for use (original, menthol, mint, nectar, fusion, melon, and tropical), all of which contained 4.8% nicotine (% w/w), glycerin, and polypropylene glycol. Participants could sample the flavors to determine which flavor they would like to use for the duration of the study. Each flavor subgroup was limited to a total of 15 participants per flavor to ensure the equal distribution of participants among the flavor groups. Once the flavor category was full, the option for that flavor was removed for the remaining participants.

### Study Procedures

At baseline, participants underwent the following assessments: physical examination, vital signs (blood pressure, heart rate, respiratory rate, and oral temperature), 12-lead electrocardiography, and clinical laboratory tests (hematology, clinical chemistry, and urinalysis). Female participants also underwent a pregnancy test.

After screening and enrollment, each participant was given a packaged Vuse Solo device, a USB charger, and sufficient e-liquid cartridges in their chosen flavor for ad libitum vaping during the 2-week study period, during which participants were able to use the product in their natural environment. The quantities of the product were assigned based on individual self-reported use before enrollment. Instructions were also provided on how to use the e-cigarette, the CME Bluetooth app on a sponsor-provided smartphone, and the PUB instrument (Figure 1). The smartphone was tested before distribution to determine that the internet connection was active, and the Bluetooth interface with the PUB instrument was established for the transfer of study data to a cloud database. The PUB instrument was attached to the e-cigarette between the power unit and the e-liquid cartridge and recorded puffing data during the periods of product activation. Puff duration, interval between puffs, total number of puffs, and battery characteristics (voltage and current data) were captured. At the end of each day, participants were requested to sync their PUB instrument with the CME Bluetooth app to transfer their puff data.

Participants were instructed to use the investigational product exclusively (they were not to use their usual brand of ENDS) but were allowed to continue the use of all other nicotine and tobacco products. Participants were contacted weekly by telephone to check that the study protocol was being followed and that they understood how to use the supplied devices. Any new medications were reported to allow the principal investigator to decide whether the participant could continue to participate in the study.

The first 2 weeks of the study were considered an acclimation period with participants being contacted by telephone to determine whether they had experienced any complaints related to the test product at the end of this continuous 14-day period. Any significant complaints were reported to the principal investigator for further evaluation and to determine whether the complaint qualified as an adverse event. The next continuous 2 weeks of the study, which began immediately after the acclimation period, constituted the product use evaluation period. Along with the topography data, product experience data were collected with the Product Evaluation Scale (PES), in which participants answered 21 questions by providing scores on a 7-point Likert scale ranging from 1=*not at all* to 7=*extremely*. The PES was adapted from the Cigarette Evaluation Questionnaire [23]. The PES response scores were aggregated into 5 domains (satisfaction, psychological reward, aversion, comfort and ease of use, and relief). This evaluation was completed at the conclusion of the study along with a repeat physical examination before the participants were discharged from the study.

### PUB Instrument

The PUB instrument (Figure 1) was designed to capture voltage and current data as well as puff duration during the puffing process for an assortment of nicotine delivery products in a natural ambulatory environment with little to no impact on product performance (Table 1). The PUB instrument used in this study was created specifically for the ENDS study product. The PUB instrument connects between the power unit and the e-liquid cartridge of the e-cigarette and is automatically activated when a participant takes a puff. Battery voltage is measured by the PUB instrument across a shunt resistor to calculate current data, and puff duration is measured by the initial time at which the voltage rises from 0 (rising edge) to the point before the voltage returns to 0 (falling edge).

**Table 1:** Comparison of Vuse Solo electronic nicotine delivery system functionality with and without the Product Use and Behavior (PUB) device attached.

Characteristics	Vuse Solo	Vuse Solo with PUB device attached
Product battery life (puffs)	350-360	350-360
PUB battery life (calculated total puffs)	N/A^a^	1000
Idle battery life (h)	>168	36
Puff range/cartridge^b^	180-200	194-214
Charging time (min)	10-60	N/A
PUB charging time (min)	N/A	15-60
Total particulate matter^b^, mean (SD)	2.34 (0.12)	2.17 (0.11)
Pressure drop (mm Wg), mean (SD)	84 (15)	105 (11)
Product length (mm)	120.5	180
Product weight (g)	15.9	27.6
Activated product voltage (V)	3.3	3.3
Coil resistance (ohms)	2.1-3.0	2.2-3.1

^a^N/A: not applicable.

^b^Products tested using a puffing regimen of 55 mL volume, 30 second interpuff interval, and a 3 second puff duration.

The PUB instrument contains an electrically erasable programmable ROM (EEPROM) chip that stores puff data (approximately 1000 puffs) and a Bluetooth chip that allows the transfer of data to a host Bluetooth device using a mobile phone app. The PUB instrument (version 1) was developed specifically for the Vuse Solo, Ciro, and Vibe e-cigarettes, with the Vuse Solo PUB instruments fitted with connectors to allow connection to the Vuse Solo battery and cartridge.

### Mobile Phone App and Provided Smartphones

The PUB Bluetooth Low Energy (BLE) transfer app was designed to allow the participants mobile device to discover the PUB instrument, receive stored puffing data from the PUB, and transfer the data to the database. The CME PUB BLE transfer app was designed to work with Windows, Android, and Apple devices. The transferred data were redirected through a secure Amazon Web Services Kinesis Data Firehose stream to the database table.

To ensure that all participants had a smartphone capable of using the CME BLE transfer app, Samsung Galaxy S8 smartphones were provided to every participant. The Android devices, provided by Stefanini IT Solutions, had the CME BLE transfer app preinstalled and had all phone features disabled, aside from those necessary for the app to work (eg, Wi-Fi and Bluetooth).

### Statistical Analysis

Assuming an SD in puff duration of 1.75 seconds [24] and that the 95% CI half-width would be no more than 0.5 second (the desired precision), it was calculated that 61 participants would be needed to give an overall probability of 85.5% (α=.05 and probability[width]=0.9) of achieving sufficient precision to capture the true mean [20]. Assuming a 20% attrition rate, the recruitment of 75 participants was deemed necessary to ensure that at least 60 (80%) would complete the study. Overall means for mean daily puffs, daily sessions, session length, IPI within sessions, puffs within sessions, mean puff duration, and intersession intervals (ISIs) were determined. A 1-way ANOVA with a post hoc analysis using Bonferroni adjustment was conducted to analyze the differences in session characteristics among low-, moderate-, and high-use participants in SPSS software (version 27.0; IBM Corp). Differences in product evaluation scores were analyzed using independent samples 2-tailed *t* tests with a Bonferroni-adjusted α level for multiple comparisons (α=.016). Of the 73 participants that completed the study, 55 were deemed appropriate for analysis. The 18 participants removed from analysis fell in the bottom 0th to 9th percentile of daily use and had fewer than 10 puffs across the 14-day product evaluation period.

### Topography and Use End Points

Time-series data for puff duration for each participant were exported to Seeq software (Seeq Corporation). First, a *capsule* (specific time periods of data) was created using the *Custom Condition* tool within Seeq to bin data from the start to the end of the participant’s data collection period. The IPI, or the time between puffs, was calculated from the end of 1 puff to the beginning of the next puff. The *Formula* tool was used within Seeq to first find and remove puffs lasting <0.5 second with an associated voltage less than the output of the device. These were considered *false puffs*, which can occur when disconnecting or connecting the device charger. Once these false puffs were removed from the puff duration signal, an IPI signal was created using the newly cleansed duration signal (refer to Formula 1 in Multimedia Appendix 1). From the IPI signal created, the upper percentiles were calculated to cleanse the data of prolonged periods of nonuse. These periods of nonuse represented the 95th percentile of intervals with a range of 12 to 16 hours of nonuse. The data cleansing step was completed by using the *Value Search* function in Seeq (refer to Formula 2 in Multimedia Appendix 1).

**Figure 2:**
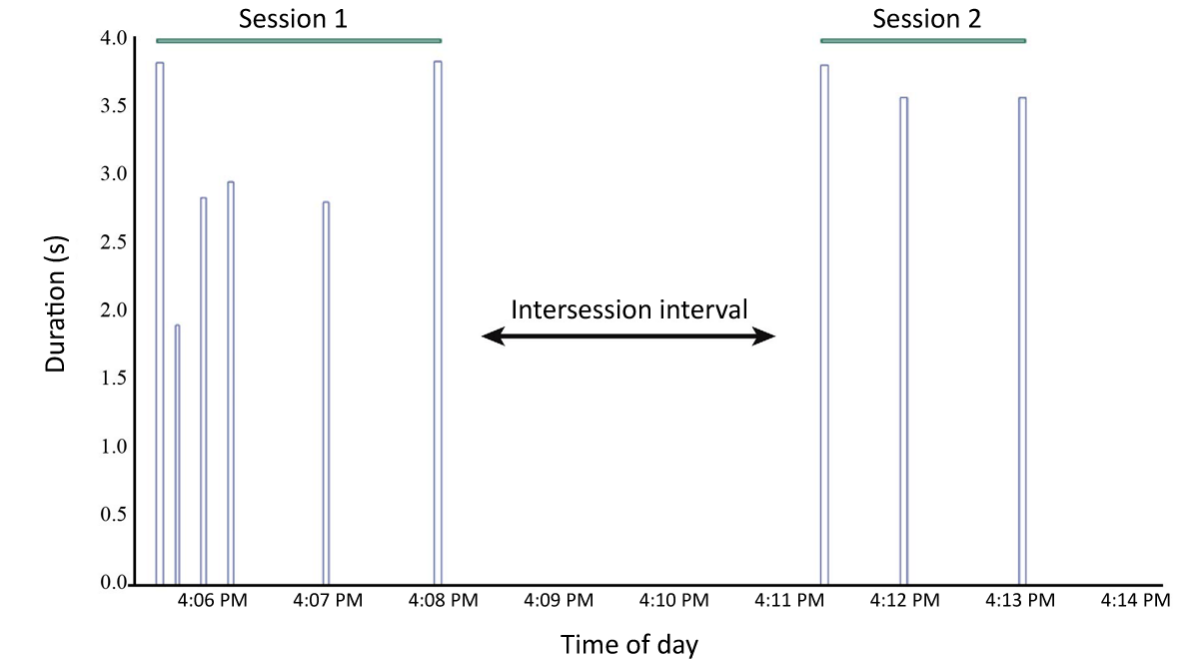
Determination of the intersession interval. The image presents puffing topography data showing the individual puffs in 2 distinct puffing sessions as well as the intersession interval between these distinct sessions.

The average IPI was then calculated with the *Signal from Condition* function within Seeq using the IPIs with long breaks removed, and sessions were determined based on an average of this IPI signal (Figure 2). Using the deviation search feature within Seeq, capsules were created for IPIs that fell above the boundary set by the average IPI and became the ISI (Figure 2). Using the *Formula* tool, sessions were created by finding the inverse of the ISI (refer to Formula 3 in Multimedia Appendix 1), which defines the end of a session as the point in time when a given participant’s IPI exceeds their mean IPI.

The final step before analysis required the creation of discrete variables for the duration data. To determine total puff time to produce the discrete variables for puff duration, the 0-second durations (time between puffs) were factored out to obtain only the actual puffing time. Using the *Formula* tool in Seeq, discrete durations were created for later analyses (refer to Formula 4 in Multimedia Appendix 1).

Because of the high amount of variability across the participant population with regard to mean daily puffs, participants were then binned into low-, moderate-, and high-use groups based on average daily use to better understand differences in participant use patterns. Participants were distribution relatively evenly across the percentile bins (10th: 5 participants, 20th: 5 participants, 30th: 9 participants, 40th: 11 participants, 50th: 5 participants, 60th: 3 participants, 70th: 9 participants, 80th: 4 participants, and 90th: 4 participants). Final analysis was conducted using the *Signal from Condition* function within Seeq; data for average session length, average puff duration within sessions, average number of puffs within sessions, average session IPI, and average ISI were assessed and exported for statistical analysis.

### Participant Use Groups

Participants’ mean daily puffs were binned into 10th percentiles with percentile ranks having mean daily puff counts as follows: 10th: 12 puffs, 20th: 23 puffs, 30th: 37 puffs, 40th: 52.8 puffs, 50th: 68.5 puffs, 60th: 89 puffs, 70th: 122.5 puffs, 80th: 160.2 puffs, and 90th: 237 puffs. Participant data were categorized into low- (12/55, 22%), moderate- (24/55, 44%), or high-use (19/55, 35%) groups based on mean daily puff percentile rankings across the 14-day product use evaluation period. Low use was defined as a mean daily puff count that fell between the 10th and 40th percentiles, moderate use was defined as a mean daily puff count that fell between the 40th and 70th percentiles, and high use was defined as a mean daily puff count that fell above the 70th percentile.

## Results

### Study Participants

A total of 75 participants were enrolled in the study. Of the 75 participants, 73 (97%) completed the study, whereas 2 (3%) were discontinued from the study early: 1 (50%) discontinued during week 1 because the PUB instrument was not working, and the participant was unable to return to the site for a replacement, whereas 1 (50%) discontinued owing to a family emergency and completed the final visit early because they were unable to complete the study on the scheduled date. The current nicotine or tobacco products being used by the 75 participants were as follows: 71 (95%) used closed-tank e-cigarettes, 4 (5%) used cigalike e-cigarettes, 31 (41%) used combustible cigarettes, 2 (3%) used filtered cigars, and 1 (1%) used open-tank e-cigarettes.

A summary of participant demographics is presented in Multimedia Appendix 2, with data shown according to the Vuse Solo ENDS flavor that the participants used in the study. Among the flavor groups, demographic characteristics, including age, weight, height, and BMI, were similar, and there were no major differences among any of the flavor or nicotine concentration groups. The proportions of male and female participants were also similar across the flavor and nicotine concentration groups. In each group, participants were predominantly non-Hispanic (74/75, 99%); overall, a majority of the participants (66/75, 88%) identified as White (Multimedia Appendix 2).

### Session Analysis

To understand participant variation across the study population, participant data were binned into percentile rankings based on daily puff counts. These percentiles were then combined to form 3 distinct groups: low- (10th to 40th percentiles), moderate- (40th to 70th percentiles), and high-use (70th to 100th percentiles) groups. A 1-way ANOVA with a Bonferroni post hoc analysis was used to compare means for the sessions of use across the different use groups. The overall mean number of daily puffs within the low-use group was 26.91 (SD 10.57), the moderate-use group had a mean daily puff count of 61.28 (SD 13.15), and the high-use group had a mean daily puff count of 184.41 (SD 97.99). These were significantly different between the low- and high-use groups (*P*<.001) and between the moderate- and high-use groups (*P*<.001) but not between the low- and moderate-use groups (*P*=.16). The session characteristics varied among the low-, moderate-, and high-use participants. Low-use participants had significantly fewer sessions per day than high-use participants (*P*<.001; Table 2). Although not significantly different, most likely owing to the high SD in the moderate-use group, low-use participants had an average of 7.49 (SD 4.68) sessions a day compared with the 30.95 (SD 41.68) of the moderate-use group (*P*=.06). When examining the average length of the sessions, moderate-use participants had shorter sessions than the low-use (*P*=.02) and high-use (*P*=.006) participants (Table 2).

**Table 2:** Puffing topography and electronic nicotine delivery system use session characteristics according to level of use^a^.

Variable	Low-use group (n=12), mean (SD)	Moderate-use group (n=24), mean (SD)	High-use group (n=19), mean (SD)	*P* value		
				Low- vs moderate-use group	Low- vs high-use group	Moderate- vs high-use group
Daily puffs	26.91 (10.57)	61.28 (13.15)	184.41 (97.99)	.16	<.001	<.001
Daily sessions	7.49 (4.68)	30.95 (41.68)	32.26 (19.56)	.06	<.001	.90
Session length (s)	203.30 (198.11)	87.86 (89.98)	212.52 (185.36)	.02	.90	.006
Interpuff interval within sessions (s)	61.66 (63.04)	25.56 (16.57)	32.78 (22.41)	.01	.08	.23
Puffs within sessions	4.40 (2.05)	3.37 (1.82)	6.71 (2.02)	.13	.004	<.001
Puff duration (s)	2.10 (0.62)	2.18 (0.71)	2.19 (0.72)	.76	.72	.93
Intersession interval (min)	255.20 (201.77)	94.09 (64.43)	84.64 (58.39)	.001	.002	.62

^a^Electronic nicotine delivery system use characteristics were grouped according to low, moderate, and high Vuse Solo electronic nicotine delivery system use.

Puffs per session were significantly different between the low- and moderate-use groups compared with the high-use group (low vs high: *P*=.004; moderate vs high: *P*<.001). There was also a significant difference between the low-use group and the moderate-use group for IPI within use sessions (*P*=.01). IPI within sessions had high SDs for the low- and high-use groups, which may suggest differences at the extreme ends of the population, which this study did not have the power to determine (Table 2).

The mean duration of each puff within sessions seemed consistent among low- (2.10, SD 0.62 s), moderate- (2.18, SD 0.71 s), and high-use (2.19, SD 0.72 s) participants, and the differences among the use groups were all not statistically significant (Table 2). The length of time between the end of a session and the beginning of the next one (ISI) varied among the use groups. The ISIs among the low-use participants were significantly longer than those among both the moderate- (*P*=.001) and high-use (*P*=.002) participants, but this was not the case between the moderate-use group and the high-use group (*P*=.62; Table 2). Overall, low-use participants had longer ISIs with fewer puffs per session and longer IPIs than the moderate- and high-use groups. Although the moderate- and high-use groups had similar IPIs and sessions per day, the high-use groups took twice as many puffs per session compared with the moderate-use group and had an average session length that was significantly longer than that of the moderate-use group.

### PES Questionnaire

The PES questionnaire was used to characterize study participant aversion, comfort and ease of use, satisfaction, psychological reward, and relief with using the study ENDS product. A Bonferroni-adjusted α level of *P*<.016 was used for significance testing to correct for multiple sampling. Participants within the high-use group reported greater ease of use with the product and were more comfortable using the ENDS product in public than those who had lower daily use (*P*=.01; Table 3). Furthermore, participants with higher daily use reported less aversion to the ENDS product than those with lower daily use (*P*=.01; Table 3). Although nonsignificant, participants with low daily use reported less satisfaction when using the ENDS product than those with higher daily use (*P*=.03; Table 3). In total, participants with higher daily use reported greater comfort using the product with less aversion than those with lower daily use, whereas feelings of psychological reward did not seem to vary.

**Table 3:** Product Evaluation Scale (PES) mean scores^a^.

PES domain	Low-use group (n=12), mean (SD)	Moderate-use group (n=24), mean (SD)	High-use group (n=19), mean (SD)	*P* value		
				Low- vs moderate-use group	Low- vs high-use group	Moderate- vs high-use group
Comfort and ease of use	4.26 (1.31)	4.38 (1.16)	5.29 (0.90)	.78	.01	.02
Satisfaction	3.97 (1.62)	4.78 (1.16)	5.05 (1.32)	.16	.03	.36
Psychological reward	3.58 (1.42)	3.98 (1.11)	3.65 (1.64)	.36	.89	.52
Aversion	2.58 (1.46)	2.22 (1.34)	1.55 (0.76)	.44	.03	.09
Relief	4.21 (1.31)	4.45 (0.90)	4.04 (1.01)	.53	.66	.23

^a^Data are presented according to the level of Vuse Solo electronic nicotine delivery system use (low, moderate, and high). Data for the single-item questions regarding ease of use and comfort using the device are combined.

## Discussion

### Principal Findings

To move toward a better understanding of topography in the context of new innovative products that may be less harmful to consumers, data collection methods and topography end points must be specific to the products being tested [4]. Conventional cigarette topography has a predefined session of use, which is inherent to the product (a single unit’s use equates to a session) and has been studied extensively [13,25-27]. Conversely, understanding the use of ENDS products extends beyond understanding a single use of the product with the context of a consumer’s normal environment removed. A number of studies have reported ENDS use characteristics in clinical settings with defined periods of use [6,28,29], but only more recently have studies been able to provide topography data from ENDS products used in an ambulatory fashion [20,30-33]. However, even in the latter studies, which began to explore ENDS use behavior in natural environments, reproducible topography end points for these novel data have proven elusive. In this study, a novel technology was used to capture data in a manner that allowed for the creation of operational definitions for ISIs and use sessions as well as to provide traditional topography end points within the context of these novel variables. Along with other recently published studies [34,35], our findings using the PUB device clearly show the strong potential of wireless connected devices in collecting puffing topography data in the real-world environment and over prolonged periods of time. In addition, our study reports, for the first time, puffing topography and use session information for the Vuse Solo ENDS, which is of importance in determining risk assessments for the use of this ENDS product and subsequently in determining its tobacco harm reduction potential.

In the data presented in this study, the stratification of daily use provides insight into the variability of how consumers use ENDS products. By creating percentile bins based on daily use, a distinction in use patterns and in the importance of various topography characteristics becomes more apparent. Participants with higher daily use took significantly more puffs per use session (6.71 vs 4.40) and puffed more frequently (IPI: 32.78 s vs 61.66 s) than participants in the low-use group (Table 2). Puff duration remained consistent across the low-, moderate-, and high-use groups (2.10 s, 2.18 s, and 2.19 s, respectively). This analytical method also allows for assessments around differences in use patterns among subsets of the study population. Although the patterns of use vary between low- and high-use groups, there is also a clear distinction when compared with the moderate-use group. The session length for the moderate-use group is 59% shorter than that for the high-use group and 57% shorter than that for the low-use group while simultaneously showing similarities to the high-use group in puff duration, ISI, IPI, and mean daily sessions. However, the number of puffs per session for the moderate-use group was similar to that for the low-use group (4.40 vs 3.37, respectively).

Finally, by stratifying across daily use and determining the daily sessions of use, it also becomes possible to use participant characterization of their product interaction to find factors that drive product use. For this particular ENDS product, comfort using the product in public and lower aversion scores were associated with a greater number of daily puffs. This type of analysis also provides an opportunity to consider questions about nicotine level, flavors, and dual or poly use of other products in future studies. With the changing regulatory landscape and the need to drive tobacco harm reduction, these questions have never been more relevant.

### Strengths and Limitations

As more is learned about ENDS products and product-specific features, it becomes important to understand new variables beyond those considered in traditional topography studies [36,37]. Even with this novel analytical approach, substantial variability can be seen within the percentile bins. This variability suggests that additional variables to characterize product use, both at category- and product-specific levels, may still need to be explored. Not only does this suggest considerations for rethinking study design to fit the product population, but it also means adapting the tools being used to collect topography data that are fit for purpose and product. Leveraging technology that allows participants to maintain normal use behavior by simplifying processes for them and collecting data in real-world settings avoids the confounds associated with modifying product-user interactions, using the product in confined conditions, and limiting the time the user has with the product [38]; for example, one of the more common devices for assessing topography in the natural environment is the Clinical Research Support System (CReSS) device [16,17,39]. Unlike the PUB instrument, this product requires additional input from the user or a laboratory technician during the initiation of each use in terms of calibrating the device, affixing it to the mouthpiece of the ENDS product, and turning it on [14-19,34,39]. The requirement for placing a measurement device between the ENDS product and the user, and the addition of further activation steps, have the potential to affect spontaneous user experience by affecting vapor output to the user; the habitual and spontaneous behaviors of use are also affected. This also gives rise to the potential alteration of puffing patterns and use behaviors, and topography assessments may reflect this difference. The use of the PUB device facilitates more naturalistic user behavior and simplifies the user experience by having the topography measurement device fixed between the battery and the e-liquid cartridge for the duration of the study period, which does not require the user to turn on and activate the device before use. Although this does allow for more natural use, there are some components of this device that still require user input. For the PUB instrument, the user is still required to upload the data collected during nightly charging sessions because instantaneous data streaming has not yet been achieved in this device. However, this method does reduce the potential for data loss during the study. In addition, another feature of the PUB device facilitates better quality data collection. Devices such as the CReSS device require adaptors to be made for each ENDS type, based on the shape of the mouthpiece of the ENDS product. These adaptors use a silicone seal to prevent air leaks during puffing, which can lead to imprecise measurements if the seal is not sufficiently tight [15]. Furthermore, although the CReSS device is adaptable to various types of ENDS products, this method of attachment to the ENDS product gives rise to a weight limitation because the use of heavier ENDS products with the CReSS device tends to create a poor flow seal or lead to the e-cigarette losing the connection completely, meaning that topography data are not accurately collected [15]. The means of attachment of the PUB device to an ENDS product therefore provides a significantly improved assessment of topography parameters compared with other commercially available devices such as the CReSS device.

One use of puffing topography data is to inform the setting of puffing parameters for the analytical testing of ENDS aerosol emissions, which in turn can be used in both absolute and relative (to cigarette smoking) risk assessments for the use of a given ENDS product. It is interesting, therefore, to assess the data collected in this study in the context of comparing the measured topography parameters with puffing regimens often stipulated for use in ENDS aerosol testing. These regimens, which use the format of puff volume:puff duration:IPI, include the International Organization for Standardization regimen (35:2:60) and the Cooperation Centre for Scientific Research Relative to Tobacco recommended method (55:3:30) [40,41]. In this study, puff volume could not be determined. However, the other topography parameters derived in this study suggest that the use of either of these defined puffing regimens could lead to the collection of ENDS emissions that do not necessarily reflect real-world use, particularly when taking into account the differences among ENDS users who use their device at a low, moderate, or high level; for example, the use of the International Organization for Standardization regimen (2-s puff duration and 60-s IPI) very closely approximates to real-world Vuse Solo ENDS use for the low-use group but not for the moderate- and high-use groups, which had a much lower mean IPI, whereas the use of the Cooperation Centre for Scientific Research Relative to Tobacco regimen would not replicate real-world use for any of the use groups. This perhaps exemplifies the importance of making puffing topography measurements and taking these assessments into account when designing ENDS emissions analysis protocols and puffing regimens.

The data presented should be interpreted in the context of some limitations. First, the study assessed puffing topography and use patterns among established ENDS users when they switched to using the Vuse Solo closed-system ENDS product. The findings may not be representative of, and generalizable to, other similar types or brands of ENDS products or of other categories of ENDSs such as closed-system and pod-based ENDS products or open-system ENDS products. In terms of generalizability, it is also noteworthy that our clinical study only assessed Vuse Solo ENDS puffing topography and use patterns in a small sample of ENDS users in a single geographic location. Larger, less geographically constrained studies are needed to mitigate this limitation. In addition, the use of the PUB instrument to assess puffing topography does not facilitate an assessment of puff volume, which is a factor used in setting machine puffing regimens when collecting ENDS aerosols for emissions analysis.

### Conclusions

Overall, in this study, the PUB device was able to track ENDS ambulatory topography in a natural setting, showing that there were differences in use patterns among low-, moderate-, and high-use participants while substantially reducing the number of user-device interactions required on the part of participants in the study. Furthermore, the analytical process used in this study allowed for a more in-depth analysis of topography characteristics that can be used to distinguish ENDSs from other forms of nicotine delivery. These findings provide valuable insight into category- and product-specific behavior that will allow for the evaluation of these products in the appropriate context and may provide more accurate input data when designing machine puffing regimens for use in Vuse Solo ENDS aerosol testing and risk assessments. Further studies may be required to confirm or extend our findings (eg, in larger populations), and the use of the PUB instrument provides a potential means of conducting such studies.

## Appendix

Multimedia Appendix 1 Formulas to calculate interpuff interval, the cleansing of interpuff interval, session calculations, and discrete durations.

Multimedia Appendix 2 Demographic characteristics of study participants for the Vuse Solo topography study.

## Abbreviations

BLE: Bluetooth Low Energy
CME: Carolina Medical Electronics
CReSS: Clinical Research Support System
e-liquid: electronic liquid
EEPROM: electrically erasable programmable ROM
ENDS: electronic nicotine delivery system
IPI: interpuff interval
ISI: intersession interval
PES: Product Evaluation Scale
PUB: Product Use and Behavior

## Data Availability

The data sets generated and analyzed during this study are available from the corresponding author on reasonable request.
