# Supplementary material for: Using a Novel Connected Device for the Collection of Puffing Topography Data for the Vuse Solo Electronic Nicotine Delivery System in a Real-World Setting: Prospective Ambulatory Clinical Study
Source: JMIR Form Res. 2023 Oct 30;7:e49876. doi: 10.2196/49876 (PMC10644193; doi:10.2196/49876)
Supplement: Multimedia Appendix 1 [file formative_v7i1e49876_app1.docx]

## Multimedia Appendix 1

**Seeq Formulas Used**

**Formula 1: Calculated Inter-puff Interval**

| **$LowDurationsToRemove = $PuffDurations.validValues().valueSearch(40h, isLessThan(0.5), 0min, isGreaterThanOrEqualTo(.5), 0min)**  **$CleansedDurations = $PuffDurations.remove($LowDurationsToRemove)**  **$CleansedDurations.transform(**  **($previous, $current, $next) -> sample(**  **$current.getKey(),**  **($next.getKey() - $current.getKey()).convertUnits('s')**  **))** |
| --- |

**Formula 2: Cleansing Inter-puff Interval**

| **$BreakPeriods = $i.validValues().valueSearch(isGreaterThan(95^th^-98^th^ Percentile))**  **$IPI. remove($BreakPeriods)** |
| --- |

**Formula 3: Session Calculation**

| **$sessions= $ISI.inverse()**  **$sessionsinstudy= $sessions.intersect($studylength)**  **$sessionsanddurations= $sessionsinstudy.union($durations)**  **$sessionsanddurations.merge(0.5s, true)** |
| --- |

**Formula 4: Discrete Durations**

| **$DurationCapules.validValues().valueSearch(isGreaterThanOrEqualTo(.5))**  **$DiscretePuffDurations.aggregate(average(), $DurationCapsules, middleKey(), 0s)** |
| --- |
