# Supplementary material for: Using a Novel Connected Device for the Collection of Puffing Topography Data for the Vuse Solo Electronic Nicotine Delivery System in a Real-World Setting: Prospective Ambulatory Clinical Study
Source: JMIR Form Res. 2023 Oct 30;7:e49876. doi: 10.2196/49876 (PMC10644193; doi:10.2196/49876)
Supplement: Multimedia Appendix 2 [file formative_v7i1e49876_app2.docx]

## Multimedia Appendix 2

Demographic Characteristics of Study Participants. Values are presented as means (standard deviation) unless otherwise indicated.

|  | **Vuse Solo Original (N=10)** | **Vuse Solo Menthol (N=10)** | **Vuse Solo Mint (N=9)** | **Vuse Solo Nectar (N=12)** | **Vuse Solo Fusion (N=12)** | **Vuse Solo Melon (N=10)** | **Vuse Solo Tropical (N=12)** | **Overall (N=75)** |
| --- | --- | --- | --- | --- | --- | --- | --- | --- |
| Age (years) | 35.3 (9.26) | 36.3 (12.98) | 38.0 (10.45) | 33.5 (14.54) | 41.4 (12.62) | 33.9 (14.16) | 37.6 (10.01) | 36.6 (12.00) |
| Weight (kg) | 99.03 (30.63) | 85.71 (18.28) | 89.50 (22.02) | 81.50 (17.08) | 88.78 (20.93) | 85.54 (25.98) | 86.50 (22.05) | 87.86 (22.12) |
| Height (cm) | 171.38 (7.13) | 172.81 (8.69) | 169.77 (7.51) | 172.50 (10.81) | 170.78 (11.46) | 168.09 (13.64) | 169.40 (9.77) | 170.71 (9.88) |
| BMI (kg/m^2^) | 33.69 (10.37) | 28.52 (4.84) | 31.02 (7.53) | 27.52 (6.27) | 30.37 (5.85) | 29.87 (6.00) | 29.96 (6.87) | 30.06 (6.90) |
| Sex [n(%)] |  |  |  |  |  |  |  |  |
| Male | 4 (40.0) | 8 (80.0) | 4 (44.4) | 6 (50.0) | 5 (41.7) | 5 (50.0) | 5 (41.7) | 37 (49.3) |
| Female | 6 (60.0) | 2 (20.0) | 5 (55.6) | 6 (50.0) | 7 (58.3) | 5 (50.0) | 7 (58.3) | 38 (50.7) |
| Ethnicity [n(%)] |  |  |  |  |  |  |  |  |
| Hispanic/Latino | 0 | 0 | 1 (11.1) | 0 | 0 | 0 | 0 | 1 (1.3) |
| Not Hispanic/Latino | 10 (100.0) | 10 (100.0) | 8 (88.9) | 12 (100.0) | 12 (100.0) | 10 (100.0) | 12 (100.0) | 74 (98.7) |
| Race [n(%)] |  |  |  |  |  |  |  |  |
| American Indian or Alaska Native | 1 (10.0) | 3 (30.0) | 1 (11.1) | 0 | 1 (8.3) | 0 | 1 (8.3) | 7 (9.3) |
| White | 8 (80.0) | 7 (70.0) | 8 (88.9) | 12 (100.0) | 11 (91.7) | 10 (100.0) | 10 (83.3) | 66 (88.0) |
| Multiple |  |  |  |  |  |  |  |  |
| American Indian or Alaska Native, White | 1 (10.0) | 0 | 0 | 0 | 0 | 0 | 1 (8.3) | 2 (2.7) |

Abbreviation: BMI, body mass index.
